# Increase in extraction of I-123 iomazenil in patients with chronic cerebral ischemia

**DOI:** 10.1371/journal.pone.0190720

**Published:** 2018-01-11

**Authors:** Hiroki Kato, Kayako Isohashi, Eku Shimosegawa, Jun Hatazawa

**Affiliations:** 1 Department of Nuclear Medicine and Tracer Kinetics, Osaka University Graduate School of Medicine, Suita, Osaka, Japan; 2 Department of Molecular Imaging of Medicine, Osaka University Graduate School of Medicine, Suita, Osaka, Japan; National Center of Neurology and Psychiatry, JAPAN

## Abstract

**Background:**

Cerebral extraction of diffusively distributed substances like oxygen has been suggested to change according to the cerebral blood flow (CBF) and status of the microvasculature. The relationships between the cerebral extraction of diffusively distributed lipophilic tracers and the severity of cerebral ischemia has not yet been clarified. In the present study, we attempted to elucidate the association between the extraction fraction of the lipophilic tracer I-123 iomazenil (IMZ) (IMZ-EF) and the oxygen extraction fraction (OEF) derived from O-15 PET in patients with chronic steno-occlusive disease of internal carotid artery (ICA) or middle cerebral artery (MCA).

**Methods:**

Seven patients with unilateral chronic severe stenosis or occlusion of the middle cerebral/internal cerebral artery were prospectively recruited for this study. All the patients underwent both O-15 PET and quantitative I-123 IMZ SPECT. Parametric images derived from the PET and SPECT scans were anatomically normalized and evaluated by automated image analysis based on the volume-of-interest template.

**Results:**

The asymmetry index (AI) of IMZ-EF was shown to be significantly correlated with the AI of OEF (r = 0.562, P < 0.001) in the internal carotid artery perfusion area. Strong and significant correlation between the AI of the influx rate constant K1 of IMZ and the AI of the cerebral metabolic rate of oxygen (r = 0.552, P = 0.001) was clarified.

**Conclusions:**

Our results suggested that the transportation efficiency of I-123 IMZ into the brain tissue was an indicator for evaluating severity of cerebral ischemia in patients with chronic steno-occlusive disease of ICA or MCA. Cerebral metabolic state can possibly be estimated by I-123 IMZ SPECT without cyclotron.

## Introduction

Extraction of diffusively distributed substances is known to change according to the cerebral blood flow (CBF) and permeability surface product (PS) of the microvasculature.[1, 2] Oxygen is one such substance, and the oxygen extraction fraction (OEF), a parameter measured in O-15 positron emission tomography (PET), reflects the metabolic reserve, and is considered to be the gold standard as a risk indicator for hemodynamic cerebral ischemia. [3] Thus, although the extraction of substances that are diffusively transported into brain is thought to change in accordance with the ischemia severity, the relationships between the cerebral extraction of lipophilic tracers and the severity of cerebral ischemia has not yet been clarified. I-123 IMZ is one of the lipophilic substances that diffuses freely into the brain tissue in a manner similar to O-15 O_2_, and binds to benzodiazepine receptors. In the present study, we examined the relationship between the extraction fraction of IMZ (IMZ-EF) and the hemodynamic risk severity in patients with chronic steno-occlusive disease of internal carotid artery (ICA) or middle cerebral artery (MCA).

## Materials and methods

### Patients

Seven patients (age: 71.9 ± 4.7 years old, male) who met the following criteria were prospectively recruited for this study in 2005 and 2006; 1) Presence of unilateral chronic atherosclerotic steno-occlusive lesions of ICA (>90% stenosis according to the North American Symptomatic Carotid Endarterectomy Trial (NASCET) criteria[4]) or MCA M1 trunk (>90% stenosis) as assessed by digital subtraction angiography (DSA); 2) time intervals between the onset of the last cerebrovascular symptoms and the SPECT of longer than one month. Patients with history of vascular reconstruction surgery, contralateral ICA or MCA stenosis (>60%) or presence of potential sources of cardiogenic embolism were excluded. (Table 1)

**Table 1:** Demographic and clinical characteristics of the patients enrolled in this study.

Case	Gender	Age	Symptom	Diagnosis
1	M	62	No symptoms	Lt. MCAO
2	M	72	Weakness in the Rt. upper extremity	Lt. ICAO, Cerebral infarction (Lt. frontal)
3	M	70	No symptoms	Rt. ICAS: 99%, Lt. ICA: 60%
4	M	73	Rt. hemiparesis (TIA)	Lt. ICAO, Rt. VAS, Lt. SAS
5	M	78	Dysarthria (TIA)	Lt. ICAS: 99%
6	M	72	Tangent dysgraphia	Lt. MCAO
7	M	76	Rt. lower extremity and Lt. upper extremity (TIA)	Lt. ICAO

ICA: internal carotid artery, MCA: middle cerebral artery, VA: vertebral artery

ICAO: ICA occlusion, MCAO: MCA occlusion, ICAS: ICA stenosis, VAS: VA stenosis, SAS: Subclavian artery stenosis

Quantitative I-123 IMZ SPECT, O-15 PET and MRI as well as DSA, were performed in all the patients within a half-year interval. This study was conducted with the approval of the Ethics Committee of Osaka University Hospital, and written informed consent was obtained from each participant of the study.

### SPECT and PET imaging

222 MBq of I-123 iomazenil was first administered by intravenous injection in each patient. SPECT imaging was performed sequentially twice (early-SPECT and delayed-SPECT) to quantify the pharmacokinetic dynamic parameters using a simplified method.[5] Venous blood sampling was performed from the antecubital vein contralateral to the injection site at 30 min after the tracer injection. Quantitative analysis was carried out based on the three-compartment two-parameter model using a single venous blood sample and a standardized arterial input function[5]. PET imaging of the CBF, OEF, cerebral metabolic rate of oxygen (CMRO_2_) and cerebral blood volume (CBV) were performed using the conventional O-15 gas steady-state method.[6] The CMRO_2_ and OEF were corrected for the measured CBV.[7] (see S1 Text for more details)

### Calculations and evaluation of the dynamic parameters

**Fig 1:**
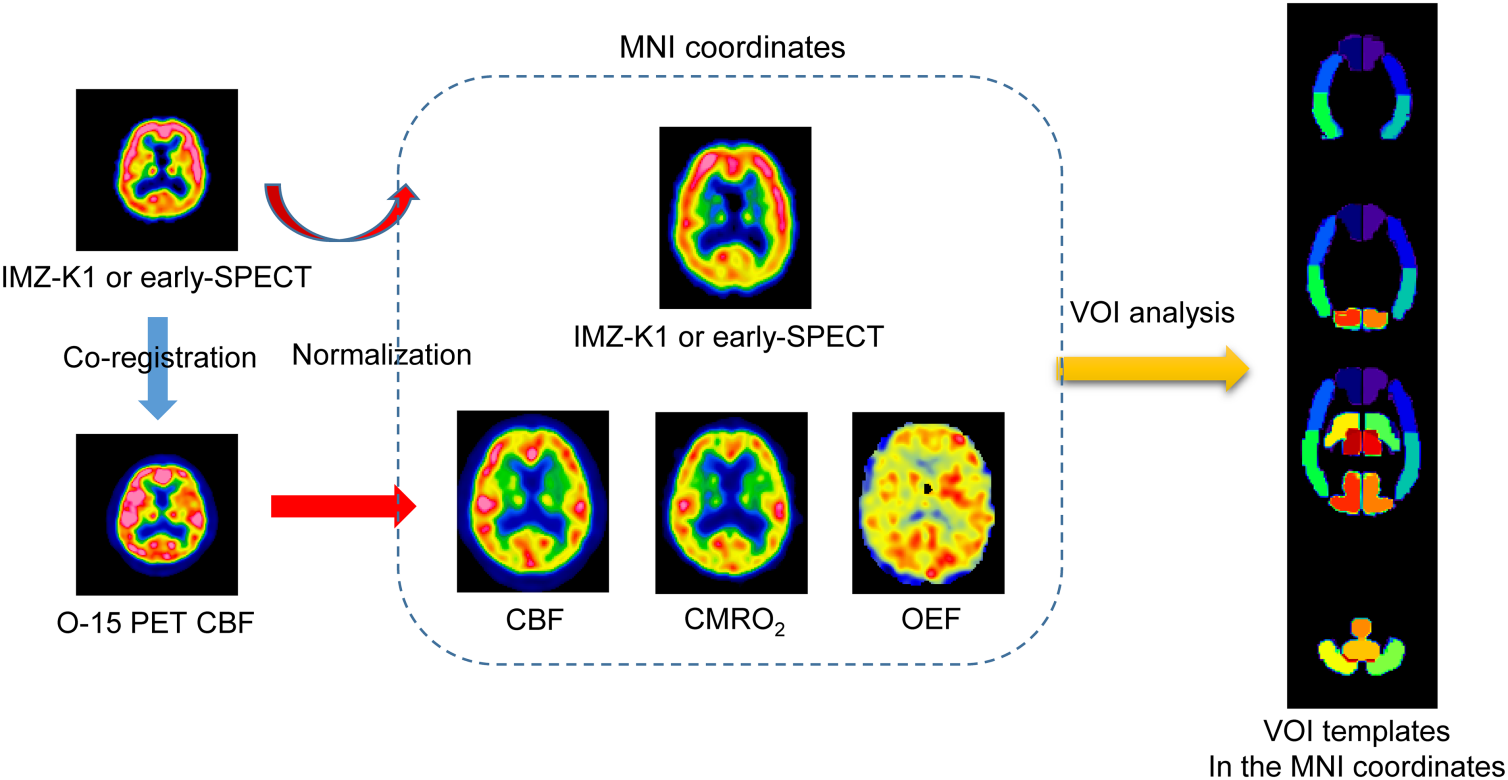
The spatial resolutions of the PET and SPECT images were first adjusted by convoluting the PET images with a 3D Gaussian function (see text). SPECT images, e.g. K1 parametric image of I-123 IMZ or early-SPECT image, were co-registered to the CBF images obtained by O-15 PET by means of linear transformation. The anatomically normalized SPECT, CBF, CMRO_2_ and OEF images were obtained by non-linear transformation using SPM8 and the PET template. IMZ-EF was calculated by dividing the IMZ-K1 by CBF. Parameters were assessed by using VOI templates in the MNI coordinates which were defined based on the brain perfusion area.

Prior to the image calculations, the spatial resolutions of the PET and SPECT images were adjusted by convoluting the PET images with a 3D Gaussian function with a FWHM of 8x8x8 mm which was determined based on the reported scanner performance tests and line profiling of the reconstructed images by ImageJ (https://imagej.nih.gov/ij/). The IMZ-EF was derived from a combination of PET and SPECT images using the following equation:

$$\text{IMZ}\;\text{-}\;\text{EF}=\frac{\text{IMZ}\;\text{-}\;\text{K1}}{\text{CBF}},$$ (1)

where IMZ-K1 is the influx rate constant K1 of the compartmental model for I-123 IMZ. The parameters of the images were evaluated by the volume of interest (VOI) located on the following 16 areas: right/left ACA, right/left MCA-M2-anterior, right/left MCA-M2-posterior, and right/left posterior cerebral artery (PCA) perfusion areas, right/left basal ganglia, right/left thalamus, right/left cerebellum, vermis, and pons. (Fig 1) (See S1 Text for more details)

The voxel value of the early SPECT images was regarded as an approximation of the IMZ-K1, because early SPECT imaging mainly represents IMZ uptake into a non-displaceable compartment. The approximating IMZ-EF (approx-IMZ-EF) was calculated as follows:

$$\text{approx}\;\text{-}\;\text{IMZ}\;\text{-}\;\text{EF}=\frac{\text{early}\;\text{-}\;\text{SPECT}}{\text{CBF}},$$ (2)

where early-SPECT represents the voxel value of early SPECT imaging.

The permeability surface area product (PS) of O_2_ (O_2_-PS) and IMZ (IMZ-PS) are calculated by the following equations:

$${\text{O}}_{\text{2}}\;\text{-}\;\text{PS}=-\text{CBF}\cdot \text{ln}\left(1-\text{EF}\right),$$ (3)

IMZ-PS=−CBF⋅ln(1−IMZ-EF). (4)

The asymmetry index (AI) for the parameter of the ipsilateral VOI A C_A_ and that of the contralateral VOI B C_B_ was calculated as follows:

$${\text{AI}}_{\left(\%\right)}=200\cdot \left({\text{C}}_{\text{A}}-{\text{C}}_{\text{B}}\right)/\left({\text{C}}_{\text{A}}+{\text{C}}_{\text{B}}\right).$$ (5)

Here VOI A/B is one of the following 5 right and left VOI pairs included in ICA or MCA perfusion area: ACA, MCA-M2-anterior, and MCA-M2-posterior perfusion areas, basal ganglia, and thalamus in the VOI templates. (Fig 1)

### Statistical analysis

The strengths of the associations were evaluated by determining the Spearman rank-order correlation coefficient using the software IBM SPSS Statistics, ver. 17.0.

## Results

**Fig 2:**
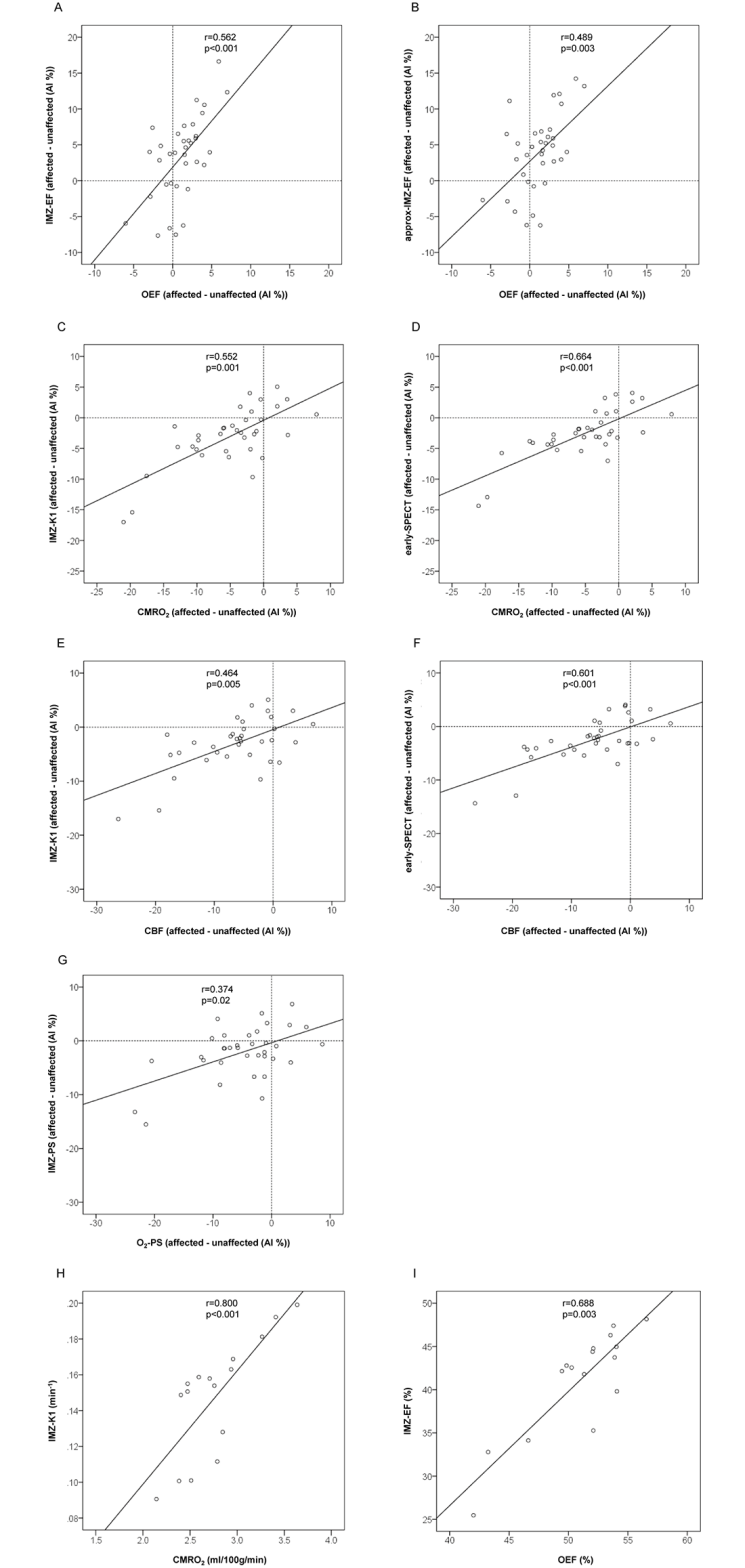
The AI of the 35 VOI pairs from the 7 patients were plotted in each graph, where the AI of each VOI value was expressed as:
${\text{AI}}_{\left(\%\right)}=\frac{200\times \left({\text{VOI}}_{\text{affected}\;\text{side}}-{\text{VOI}}_{\text{unaffected}\;\text{side}}\right)}{{\text{VOI}}_{\text{affected}\;\text{side}}+{\text{VOI}}_{\text{unaffected}\;\text{side}}}.$ Here, in this study, the “affected side” was left in the case 1, 2, 4–7, and right in the case 3 (Table 1), and the “unaffected side” was contralateral to the “affected side”. Significant correlations were found between (A) the AI of IMZ-EF and the AI of OEF, (B) the AI of approx IMZ-EF and the AI of OEF, (C) the AI of IMZ-K1 and AI of CMRO_2_, (D) the AI of early-SPECT and the AI of CMRO_2_, (E) the AI of IMZ-K1 and the AI of CBF, (F) the AI of early-SPECT and the AI of CBF, (G) the AI of IMZ-PS and the AI of O_2_-PS, (H) the AI of IMZ-K1 and the CMRO2, and (I) the AI of IMZ-EF and the OEF.

**Fig 3:**
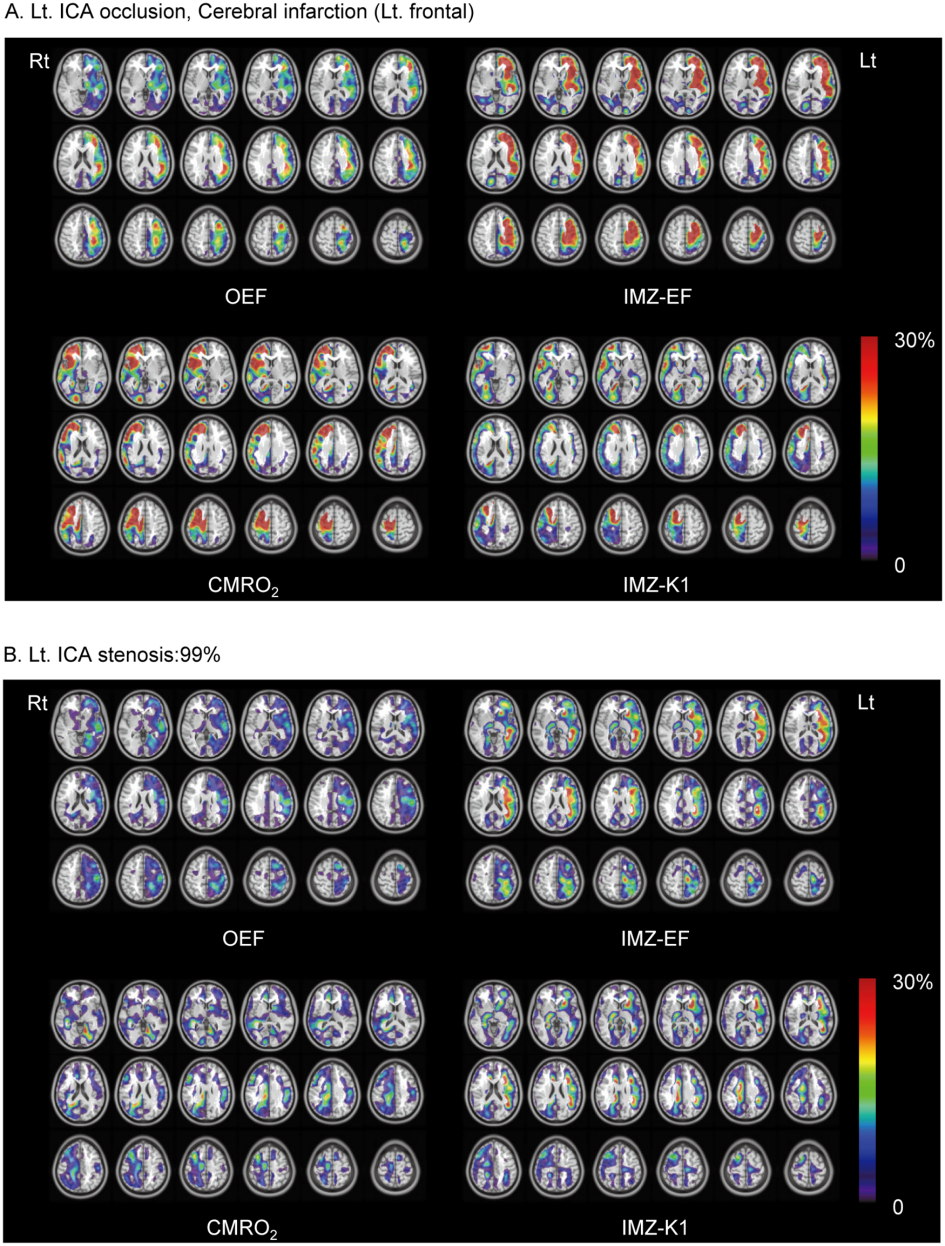
Asymmetry maps of (A) the patient with left ICA occlusion accompanied by old cerebral infarction in the left medial frontal lobe, and (B) the patient with near-occlusion of the left ICA. Asymmetry maps were created according to the following equation:
${\text{A}}_{\text{i}}{}_{\left(\%\right)}=200\cdot \left({\text{P}}_{\text{i}}-{\text{P}}_{\text{j}}\right)/\left({\text{P}}_{\text{i}}+{\text{P}}_{\text{j}}\right).$ Here A_i_ was the value of voxel i in the asymmetry map, and P_i_ or P_j_ represented the value of the voxel i or j in the parametric image respectively, where j indicated the contralateral location to i. Only positive A_i_ was displayed by the color scale and overlaid on the corresponding voxels of the symmetric MRI template. Infarcted lesions and areas where the kinetic parameter K1 could not be calculated because of noise or an extraordinarily low count of delayed SPECT images were masked.

The AI of the IMZ-EF and the approx-IMZ-EF were found to be significantly correlated with the AI of OEF. The AIs of the IMZ-K1 and the early-SPECT count also showed significant correlation with the AI of CMRO_2_. (Fig 2A–2D) The AI of IMZ-PS was found to be significantly correlated with the AI of O_2_-PS. (Fig 2G) The mean IMZ-PS and mean O_2_-PS in the MCA area of the intact hemispheres of the subjects were almost the same; 0.21 ± 0.11 ml/min/g and 0.22 ± 0.02 ml/min/g, respectively. The AI maps demonstrated that the asymmetry patterns of the IMZ-EF and the IMZ-K1 images were similar to those of the OEF and CMRO_2_ images, respectively. (Fig 3)

## Discussion

In the present study, the IMZ-EF was found to be significantly correlated with the OEF derived from O-15 PET. A significant and strong correlation between influx rate constants of I-123 IMZ (the IMZ-K1 and/or the early-SPECT count) and CMRO_2_ was also observed in these patients. I-123 IMZ is a lipophilic substance that diffuses freely into the brain tissue like O-15 O_2_ and binds to benzodiazepine receptors, thereby indicating the viability of neurons.[8, 9] Basically, the EF of lipophilic molecules has been known to change in proportion to PS and in reverse proportion to the CBF. Although the OEF changes to compensate the imbalance between the supply and the metabolic demand of oxygen in brain, it probably is controlled by some sort of regulatory mechanism attributed to cerebral capillary.

Cerebral capillary flow velocities are remarkably heterogeneous in the normal, resting state [10]. In such situations, because of “functional shunting”[11] of oxygen or IMZ, their PS and EF remain low. When the oxygen demand becomes higher, on the other hand, the capillary flow velocities become more homogeneous[12] to increase the PS, and this facilitates the extraction of oxygen,[13] IMZ or other diffusively transported substances. In the present study, actually, the O_2_-PS and IMZ-PS showed significant mutually positive correlations, and both were higher in the ischemic hemisphere than in the contralateral one. Thus, the EF of oxygen and IMZ may be controlled in similar manners by the capillary flow patterns.

In this study, the IMZ-K1 and early-SPECT showed strong and significant correlations with the CMRO_2_ (Fig 2A and 2B), and the AI map pattern of IMZ-K1 was similar to that of CMRO_2_. (Fig 3) These correlations were stronger than the well-known correlation observed between IMZ-K1 and the CBF. (Fig 2C) CMRO_2_ was calculated by the following equation:

$${\text{CMRO}}_{2}=\text{OEF}\cdot \text{CBF}\cdot {\text{S}}_{\text{a}}{\text{O}}_{2},$$ (6)

where S_a_O_2_ is the saturation of oxygen in the arterial blood. Eq (1) can be rewritten as follows:

IMZ-K1=IMZ-EF⋅CBF. (7)

The above-described correlations can be explained by Eqs (6) and (1) and the significant correlation between IMZ-EF and the OEF.

Although CBF SPECT using I-123 iodoamphetamine, Tc-99m hexamethyl propylene amine oxime or Tc-99m ethyl cysteinate dimer (ECD) has been known to be a reliable tool for in vivo measurement of the CBF, these tracers are lipophilic and the relationships between their uptake and CBF are possibly affected at least to some extent by the status of the microvasculature or capillaries. Actually, it has been reported that the brain uptake of Tc-99m ECD at more than 15 min after the injection was more strongly associated with the CMRO_2_ than the CBF in patients with cerebral ischemic diseases.[14]

The present study had some limitations. First, the accuracy of the statistical processing was not adequate because of the small number of subjects in this preliminary study. Second, the two different modalities of O-15 PET and I-123 IMZ SPECT were used to access the IMZ-EF in order to evaluate both the CBF and OEF. Therefore, the estimated IMZ-EF and IMZ-PS contained position-dependent error, because the spatial resolution, effect of attenuation or scatter, and characteristics of the artifacts were different between the two modalities. In this study, we evaluated the correlations based on raw parameter value of PET or SPECT in the 7 × 16 VOIs (Fig 1) as well as AI (Fig 2). As a result, the correlations based on raw parameter value between IMZ-EF and OEF (r = 0.365, p<0.001) and between IMZ-K1 and CMRO_2_ (r = 0.428, p<0.001) were shown to be statistically significant but weaker than those based on the AI (Fig 2A and 2C). The correlations may possibly be affected by the error in parameter estimation by using the two different modalities, which might be partially compensated by using AI.

In conclusion, a significant correlation between the extraction of I-123 IMZ and the OEF were demonstrated in patients with chronic steno-occlusive disease of ICA or MCA. Cerebral metabolic state can possibly be estimated by I-123 IMZ SPECT without cyclotron.

## Supporting information

S1 Text S1-Text.docx: Supporting Information. (DOCX)
