# Supplementary material for: Increase in extraction of I-123 iomazenil in patients with chronic cerebral ischemia
Source: PLoS One. 2018 Jan 11;13(1):e0190720. doi: 10.1371/journal.pone.0190720 (PMC5764280; doi:10.1371/journal.pone.0190720)
Supplement: S1 Text — (DOCX) [file pone.0190720.s001.docx]

**Supporting Information**

**Supplemental Methods**

**SPECT imaging**

Early SPECT imaging was started immediately after the injection and continued for 36 minutes, and delayed SPECT imaging was begun 162 minutes after the injection, so that the mid-scan time of the delayed scan was at 180 min after the injection. Venous blood sampling was performed from the antecubital vein contralateral to the injection site at 30 min after the tracer injection. The SPECT scanning was carried out using a SPECT camera[[1](#_ENREF_1)] (Gamma View SPECT 2000H; Hitachi Medical Corp., Tokyo, Japan) with four gamma-ray detectors connected by low-energy middle-resolution thin-section parallel-hole collimators (LEMR). SPECT images were reconstructed by the filtered back projection method, and the attenuation correction was performed using Chang’s method,[[2](#_ENREF_2)] with an optimized effective attenuation coefficient of 0.12 cm^-1^. Quantitative analysis was carried out by the minimally-invasive table look-up method, based on the three-compartment two-parameter model using a single venous blood sample and a standardized arterial input function.[[3](#_ENREF_3)] Briefly, parameters K1/k2 and k4 were hypothesized to be 3.00 and 0.26 respectively in this study. Based on these parameters and the arterial input function estimated by venous sampling, two look-up tables were generated, in which K1 and k3 were uniquely determined. Parametric images of K1 and k3 were generated by using in-house software, similar to the one used in our previous study.[[4](#_ENREF_4)]

**PET imaging**

PET scanning was performed using the Headtome V/SET 2400W system (Shimadzu Co., Ltd., Kyoto, Japan). Before the emission scan, a transmission scan was obtained for 10 minutes with the Ge-68/Ga-68 built-in radiation source for attenuation correction. The resolution of all the scans was 3.7 mm full width at half maximum (FWHM) in the transaxial direction and of 5 mm FWHM in the axial direction. All tomographic images were reconstructed using an ordered subset expectation maximization algorithm (12 iterations with 4 ordered subsets). The head of each subject was fixed in a head holder and positioned using laser beams so that transaxial slices parallel to the OM line could be obtained. Reconstructed images were formatted as a 3D dataset with 63 slices (3.17 mm thick) in 128x128 matrices.

**MRI**

MRI scans were acquired using the following scanners: MAGNETOM VISION 1.5T (Siemens AG, Erlangen, Germany), SIGNA EXCITE 1.5T (GE Yokogawa Medical Systems Ltd., Tokyo, Japan), or GENESIS SIGNA 1.5T (GE). T2-weighted 2D fast spin echo sequences were included in the protocol: (scan parameters: axial plane; FOV 250 mm; matrix 256 x 256 or 512 x 512; slice thickness 5 mm; interslice gap 1 to 1.5 mm; TE: 90 to 131 ms; TR: 4500 to 5000 ms).

**Automated volume of interest (VOI) analysis**

Prior to the VOI analysis, each early SPECT image was spatially normalized by SPM8 (<http://www.fil.ion.ucl.ac.uk/spm/software/spm8/>) to the I-123 IMZ SPECT template created from the normalized I-123 IMZ SPECT images of healthy volunteers.[[4](#_ENREF_4)] Delayed SPECT images and the parametric images were also normalized using the same normalization matrix as that generated in the above-mentioned normalization process for the early SPECT images. PET parametric images were also normalized to the PET template of SPM8. VOI analysis was automatically performed for these normalized images by using an in-house tool with normalized VOI templates.[[4](#_ENREF_4)] The VOI template based on the blood vessel regions bundled with the software voxel-based stereotactic extraction estimation (vbSEE) [[5](#_ENREF_5)] was transformed to MNI coordinates and used as the template for the VOI analysis (Fig. 1).

**Asymmetry Index Map**

The transformation matrices of the non-linear co-registration from the NMI 3D T1WI template of SPM8 to the symmetric 3D TIWI template (<http://www.bic.mni.mcgill.ca/ServicesAtlases/ICBM152NLin2009>) were produced by SPM8. The normalized images described in the previous subsection were non-linearly co-registered to the symmetric 3D TIWI template based on the transformation matrices. The asymmetry index (AI) map was produced by voxel-by-voxel calculation from each pair of symmetric images and the flipped versions.

**Supplemental References**

1. Kimura K, Hashikawa K, Etani H, Uehara A, Kozuka T, Moriwaki H, et al. A new apparatus for brain imaging: four-head rotating gamma camera single-photon emission computed tomograph. J Nucl Med. 1990;31(5):603-9. PubMed PMID: 2341896.

2. Chang LT. A method for attenuation correction in radionuclide computed tomography. IEEE Trans Nucl Sci 1978;25:638-43.

3. Onishi Y, Yonekura Y, Nishizawa S, Tanaka F, Okazawa H, Ishizu K, et al. Noninvasive quantification of iodine-123-iomazenil SPECT. J Nucl Med. 1996;37(2):374-8. PubMed PMID: 8667079.

4. Kato H, Shimosegawa E, Isohashi K, Kimura N, Kazui H, Hatazawa J. Distribution of cortical benzodiazepine receptor binding in right-handed healthy humans: a voxel-based statistical analysis of iodine 123 iomazenil SPECT with partial volume correction. AJNR Am J Neuroradiol. 2012;33(8):1458-63. PubMed PMID: 22403779.

5. Uruma G, Kakuda W, Abo M. Changes in regional cerebral blood flow in the right cortex homologous to left language areas are directly affected by left hemispheric damage in aphasic stroke patients: evaluation by Tc-ECD SPECT and novel analytic software. European journal of neurology. 2010;17(3):461-9. Epub 2009/11/20. doi: 10.1111/j.1468-1331.2009.02849.x. PubMed PMID: 19922460.
